# Opportunistic Bone Health Assessment Using Contrast-Enhanced Abdominal CT: A DXA-Referenced Analysis in Liver Transplant Recipients

**DOI:** 10.3390/diagnostics16010029

**Published:** 2025-12-22

**Authors:** Nurullah Dag, Hilal Er Ulubaba, Sevgi Tasolar, Mehmet Candur, Sami Akbulut

**Affiliations:** 1 Department of Radiology, Faculty of Medicine, Inonu University, Malatya 44280, Türkiye; hilal.er@inonu.edu.tr (H.E.U.); sevgi.tasolar@inonu.edu.tr (S.T.); mehmet.candur@inonu.edu.tr (M.C.); 2 Department of Surgery and Liver Transplant Institute, Faculty of Medicine, Inonu University, Malatya 44280, Türkiye; akbulutsami@gmail.com

**Keywords:** liver transplantation, bone health, computed tomography, Hounsfield unit, dual-energy X-ray absorptiometry

## Abstract

**Objective:** This study aimed to investigate the relationship between computed tomography (CT)-derived Hounsfield Unit (HU) measurements and dual-energy X-ray absorptiometry (DXA) and to evaluate the feasibility of using contrast-enhanced abdominal CT as a complementary tool in the assessment of bone health in liver transplant recipients. **Methods:** This retrospective descriptive and analytical study included adult liver transplant recipients who underwent both contrast-enhanced abdominal CT and DXA within a three-month interval. HU measurements were obtained from sagittal and axial reformatted images at the lumbar spine (L1–L4) and femoral neck. All CT examinations were performed using a standardized venous-phase protocol. DXA-derived T-scores from the lumbar spine and femur served as the reference standard. Correlation analyses and receiver operating characteristic (ROC) curves were used to evaluate associations between HU values and BMD, as well as the diagnostic performance of HU in identifying low bone density. **Results:** A total of 259 recipients (mean age 55.7 ± 14.4 years; 62.9% male) were included. Based on lumbar spine DXA, 17.8% had normal BMD, 36.7% were osteopenic, and 45.5% were osteoporotic. CT-derived HU values at both the lumbar spine and femoral neck were significantly lower in patients with reduced BMD and showed a graded decline across worsening DXA categories. HU values demonstrated positive correlations with corresponding T-scores. Diagnostic performance for detecting osteoporosis was fair, with AUCs of 0.700 (sagittal), 0.698 (axial), and 0.751 (femoral). **Conclusion:** Contrast-enhanced abdominal CT provides useful ancillary information for opportunistic bone health assessment. CT-derived HU values offer a rapid and cost-effective complementary tool but should not replace DXA in the diagnostic evaluation of osteoporosis

## 1. Introduction

Since the first successful liver transplantation (LT) performed by Starzl et al. [1] in 1967, LT has evolved into an established, life-saving therapeutic option not only for patients with end-stage liver disease and hepatic malignancies, but also for selected cases of acute liver failure and a broad spectrum of pediatric metabolic disorders [2,3]. Advances in surgical techniques, perioperative management, and modern immunosuppressive regimens have collectively contributed to the marked improvement in post-transplant survival, enabling substantial gains in graft function, long-term outcomes, and overall patient prognosis [4,5]. As post-transplant survival continues to improve, the prevention, early detection, and effective management of long-term complications have become increasingly critical due to their significant impact on recipients’ functional capacity and overall quality of life [6]. Among these late complications, skeletal disorders—particularly osteoporosis and fracture risk—constitute a major yet frequently under-recognized source of morbidity in LT recipients [7,8].

Osteoporosis in LT recipients stems from a multifactorial pathophysiology involving both pre-existing metabolic disturbances of chronic liver disease and post-transplant factors [9]. Nearly 75% of patients with chronic liver disease exhibit osteoporosis and/or fragility fractures—often referred to as hepatic osteodystrophy—reflecting a substantial skeletal burden even before LT [10]. Prior to LT, long-standing cholestasis contributes to osteoblast dysfunction through the accumulation of bilirubin and bile acids, which also impair the absorption of fat-soluble vitamins D and K, both essential for bone remodeling. Additionally, chronic liver disease is characterized by growth hormone resistance, reduced IGF-1 levels, diminished osteoblastic signaling, and altered vitamin D metabolism, including attenuated hepatic hydroxylation and enhanced degradation via CYP24A1, all of which exacerbate bone loss [10,11,12]. After LT, exposure to high-dose glucocorticoids, calcineurin inhibitors, and periods of immobilization can further accelerate bone demineralization, with the steepest decline typically occurring within the first postoperative year [10,11,13]. Given that fragility fractures markedly impair mobility, functional capacity, and quality of life, early identification and timely management of post-transplant osteoporosis are indispensable components of long-term survivorship care [14].

Dual-energy X-ray absorptiometry (DXA) remains the reference standard for assessing bone mineral density (BMD) and diagnosing osteoporosis in adult patients [15,16]. Although DXA provides well-validated quantitative measurements, its use may be constrained in clinical follow-up by limited availability and the need for separate scheduling, particularly in transplant recipients who already undergo intensive imaging surveillance. In contrast, abdominal computed tomography (CT) is routinely utilized in the post-transplant setting to assess the anatomy of the transplanted graft, evaluate vascular patency, and detect both early postoperative and long-term complications [17,18]. This frequent imaging creates an opportunity to obtain lumbar vertebral Hounsfield Unit (HU) measurements from existing scans, offering a practical and cost-effective means of estimating bone density without additional radiation exposure. Previous studies [19,20,21] in adults have demonstrated a correlation between CT-derived HU values and DXA-based BMD; however, these investigations have largely relied on non-contrast CT protocols specifically targeted to the lumbar spine. To our knowledge, no study has evaluated the opportunistic use of contrast-enhanced abdominal CT in adult liver transplant recipients for osteoporosis. Unlike earlier work in non-transplant cohorts, our study leverages venous-phase contrast-enhanced abdominal CT acquired during routine post-transplant surveillance and provides transplant- and protocol-specific HU reference values in this high-risk population. Therefore, the aim of this study was to investigate the relationship between lumbar vertebral HU measurements obtained from routine contrast-enhanced abdominal CT and DXA in adult LT recipients, exploring the feasibility of CT as a complementary tool for opportunistic evaluation of bone health.

## 2. Materials and Methods

### 2.1. Study Design and Population

This descriptive, analytic, and retrospective single-center study evaluated adult patients (≥18 years) who underwent LT between January 2012 and July 2024. Patients were included if they had undergone contrast-enhanced abdominal CT within a three-month interval before or after DXA examination. Individuals with congenital or acquired deformities of the vertebrae or femur, focal bone lesions such as cysts or metastases, a history of spinal or hip surgery involving metallic implants, or severe motion or beam-hardening artifacts that interfered with measurement accuracy were excluded. Patient selection was therefore driven by CT image quality and measurability, and only after this CT-based screening were the corresponding DXA examinations retrieved, ensuring that all included cases had analyzable lumbar DXA measurements.

### 2.2. CT Acquisition Protocol and HU Measurements

**Figure 1:**
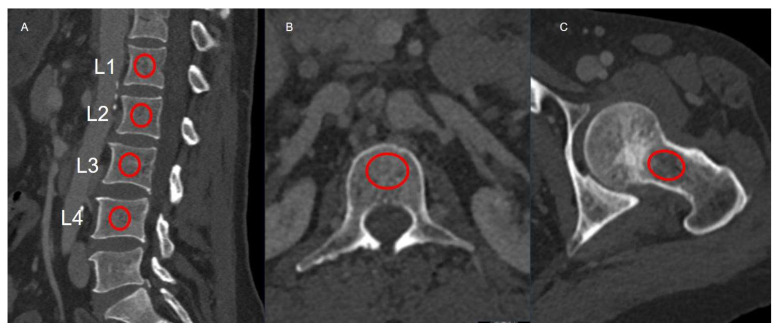
Sagittal lumbar (**A**), axial lumbar (**B**), and femoral neck (**C**) CT images demonstrating regions of interest (red circles) used for HU measurements. The ROIs shown are for demonstration purposes only; actual quantitative measurements were performed using workstation-defined ovoid ROIs with a diameter of approximately 2–2.5 cm.

All CT examinations were performed as part of routine post-transplant surveillance using multi-detector scanners, including the Somatom Definition 256-slice system (Siemens Healthineers, Erlangen, Germany) and the Aquilion 64-slice system (Canon Medical Systems, Otawara, Japan; formerly Toshiba Medical Systems), during the venous phase after intravenous contrast administration. Imaging parameters were standardized across scanners: tube voltage of 120 kVp, automated tube current modulation, 1 mm slice thickness, and a 512 × 512 reconstruction matrix. Images were reconstructed using a standard soft-tissue convolution kernel in both axial and sagittal planes to provide optimal delineation of trabecular bone. Quantitative attenuation measurements were independently performed by two radiologists, each with more than ten years of experience in abdominal imaging, blinded to the DXA findings. Circular regions of interest (ROIs) were manually placed within the central trabecular portion of the L1–L4 vertebral bodies in both axial and sagittal reconstructions, carefully avoiding cortical bone, endplates, and adjacent vascular structures to ensure consistent trabecular sampling. For femoral measurements, the ROI was placed within the medial trabecular region of the femoral neck, just inferior to the physeal scar, with care taken to exclude cortical bone and the superior margin that is particularly susceptible to partial-volume effects. For each site, the mean HU value was recorded, and the average of L1–L4 measurements was used as the representative vertebral attenuation for subsequent analysis. Lumbar HU measurements were made in both sagittal and axial planes. Femoral HU measurements were performed only from the left side, consistent with DXA examination (Figure 1).

### 2.3. DXA Examination and Classification

Bone mineral density was assessed using a DXA system (Primus, Osteosys Co., Ltd., Guro-gu, Seoul, Republic of Korea) at both the lumbar spine (L1–L4) and femoral neck. Results were expressed as T-scores and classified according to the World Health Organization (WHO) criteria: normal (T-score ≥ −1.0), osteopenia (−1.0 >T-score > −2.5), and osteoporosis (T-score ≤ −2.5) [22]. All DXA examinations were performed by technologists following the manufacturer’s calibration and quality-control procedures.

### 2.4. Study Groups

Patients were compared across three analytical frameworks. First, in accordance with WHO criteria and previous osteoporosis-related studies, patients were stratified into two age-based groups: <60 years and ≥60 years. These groups were compared with respect to radiological, and DXA-derived measurements. Second, based on lumbar spine T-scores obtained by DXA, patients were categorized into normal, osteopenic, and osteoporotic groups. These categories were compared according to radiological characteristics and DXA-derived measurements. Third, a correlation analysis was performed between quantitative measurements obtained from CT and those derived from DXA.

### 2.5. Study Protocol, Ethical Approval, and Funding

This study was conducted in accordance with the ethical principles of the Declaration of Helsinki (1964) and its subsequent amendments, and complied with all applicable institutional and national regulations governing research involving human participants. Ethical approval was obtained from the Inonu University Institutional Review Board (IRB) for Non-Interventional Studies (Approval Year: 2025; IRB No: 6312). As the study had a retrospective design involving review of medical records and re-evaluation of archived radiological images, the requirement for individual informed consent was waived. The research was financially supported by the Inonu University Scientific Research Projects Coordination Unit (Grant No: 2025/4387). The design, conduct, analysis, and reporting of the study adhered to the Strengthening the Reporting of Observational Studies in Epidemiology (STROBE) guidelines to ensure methodological rigor and minimize potential sources of bias [23].

### 2.6. Statistical Analysis

Statistical analyses were conducted using SPSS version 22.0 (IBM Corp., Armonk, NY, USA). Data distribution was assessed with the Shapiro–Wilk test. Continuous variables were expressed as mean ± standard deviation (SD). The relationship between CT-derived HU values and DXA T-scores was evaluated using Pearson’s correlation coefficient, with correlation strength interpreted as negligible (0.0–0.20), weak (0.21–0.40), moderate (0.41–0.60), strong (0.61–0.80), or very strong (0.81–1.00) [24]. Diagnostic performance of vertebral and femoral HU values for detecting low BMD was analyzed using receiver operating characteristic (ROC) curve analysis. The area under the curve (AUC), optimal HU cut-off points, and corresponding sensitivity and specificity with 95% confidence intervals (CIs) were calculated. A *p*-value below 0.05 was considered statistically significant. A post hoc power analysis based on the observed correlation between sagittal HU and lumbar DXA T-score (r = 0.44; *n* = 259) demonstrated >99% power at α = 0.05.

## 3. Results

### 3.1. Demographic and Clinical Characteristics of Entire Cohort

A total of 259 adult liver transplant recipients were included in the study, with a mean age of 55.7 ± 14.4 years; 134 patients (51.7%) were younger than 60 years, and 125 (48.3%) were aged 60 years or older. Males accounted for 62.9% (*n* = 163) of the study population. The mean interval between CT and DXA examinations was 0.97 ± 1.92 months (approximately 29 ± 58 days), with a minimum of 15 days and a maximum of 89 days. The mean lumbar spine BMD was 0.80 ± 0.13 g/cm^2^, corresponding to a mean T-score of −2.37 ± 1.45. Based on lumbar spine DXA classification, 17.8% of patients were within the normal range, 36.7% were osteopenic, and 45.5% were osteoporotic. The mean femoral BMD was 0.85 ± 0.18 g/cm^2^, and the corresponding T-score averaged −0.74 ± 1.24, with 71.8% of participants classified as normal, 17.8% osteopenic, and 10.4% osteoporotic (Table 1).

### 3.2. Quantitative CT Measurements and Age-Related Differences

**Figure 2:**
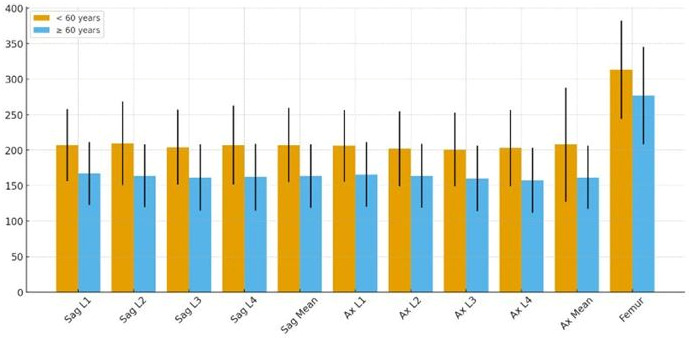
Comparison of lumbar and femoral HU measurements between patients aged <60 years and ≥60 years. All quantitative CT parameters—including sagittal mean HU, axial mean HU, and femoral HU—were significantly lower in patients aged ≥60 years compared with those aged <60 years (*p* < 0.05 for all comparisons).

Mean vertebral HU values measured in both sagittal and axial planes demonstrated a significant age-related decline. Patients younger than 60 years had higher sagittal mean HU values (207 ± 52.5) compared with those aged 60 years or older (164 ± 44.8; *p* < 0.001). A similar pattern was observed in axial mean HU (208 ± 80.5 vs. 162 ± 44.5; *p* < 0.001). Femoral HU values also showed a significant reduction in the older age group (313 ± 69.2 vs. 277 ± 68.8; *p* < 0.001). However, no statistically significant differences were found in lumbar or femoral BMD and T-scores between age groups (*p* > 0.05 for all) (Table 2, Figure 2).

### 3.3. Relationship Between HU Values and DXA-Based Bone Status

Both vertebral and femoral HU values differed significantly across DXA-based lumbar T-score categories (normal, osteopenic, osteoporotic; ANOVA *p* < 0.001). The sagittal mean HU was highest in the normal BMD group (216 ± 54.3) and progressively decreased in osteopenic (197 ± 45.1) and osteoporotic patients (165 ± 51.3). Post hoc analyses revealed significant differences between normal and osteoporotic (*p* < 0.001) and between osteopenic and osteoporotic groups (*p* < 0.001). Similar trends were observed for axial HU values (normal: 214 ± 55.5; osteopenic: 200 ± 86.3; osteoporotic: 163 ± 49.7; *p* < 0.001). Femoral HU values also decreased progressively: normal (307 ± 69.7), osteopenic (279 ± 68.9), and osteoporotic patients (239 ± 58.5; *p* < 0.001) (Table 3).

### 3.4. Correlation Between Quantitative CT and DXA Parameters

**Figure 3:**
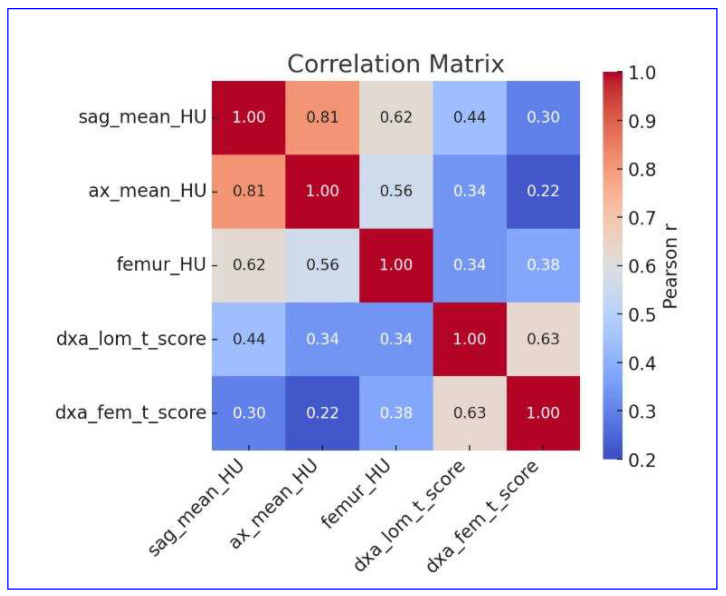
Correlation matrix showing Pearson correlation coefficients between CT-derived HU measurements and DXA T-scores.

A significant positive correlation was found between sagittal mean HU and both lumbar and femoral DXA T-scores (r = 0.444 and r = 0.301, respectively; *p* < 0.001). Axial mean HU values also correlated with lumbar (r = 0.338, *p* < 0.001) and femoral (r = 0.219, *p* < 0.001) T-scores. Femoral HU values showed moderate correlations with lumbar (r = 0.342, *p* < 0.001) and femoral (r = 0.375, *p* < 0.001) T-scores (Figure 3).

### 3.5. Diagnostic Performance of CT-Derived HU Values

**Figure 4:**
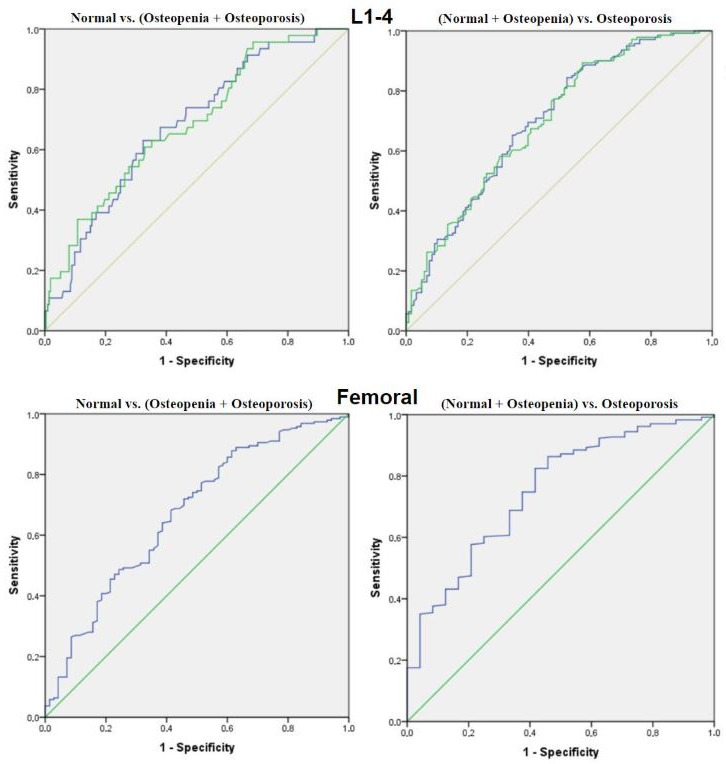
ROC curves illustrating the diagnostic performance of lumbar (L1–L4) and femoral HU measurements for detecting low bone mineral density.

ROC analysis demonstrated moderate diagnostic performance of CT-derived HU values for identifying low bone mineral density. Distinguishing normal bone from osteopenia/osteoporosis, the AUC was 0.682 (95% CI: 0.601–0.763; *p* < 0.001) for sagittal HU and 0.686 (95% CI: 0.603–0.768; *p* < 0.001) for axial HU, with optimal cut-off values of 203 HU and 205 HU, respectively. The femoral HU yielded an AUC of 0.670 (95% CI: 0.595–0.746; *p* < 0.001) and a cut-off value of 240 HU (sensitivity = 0.83; specificity = 0.57). When differentiating osteoporotic patients from those with normal or osteopenic bone density, the diagnostic accuracy improved slightly, with AUCs of 0.700 for sagittal HU, 0.698 for axial HU, and 0.751 for femoral HU. The optimal femoral HU threshold of 237 HU yielded 82% sensitivity and 63% specificity (Table 4, Figure 4).

## 4. Discussion

In the present study, it was demonstrated that quantitative HU measurements obtained from routine contrast-enhanced abdominal CT scans are significantly associated with DXA-derived bone mineral status in adult liver transplant recipients. Vertebral and femoral HU values were observed to decrease progressively from normal to osteopenic and osteoporotic groups, and positive correlations were identified between these values and both lumbar and femoral T-scores. Taken together, these findings indicate that opportunistic bone health assessment can be feasibly performed using CT examinations already obtained as part of standard post-transplant surveillance. In addition to confirming the feasibility of HU-based assessment in this population, our study provides venous-phase, contrast-enhanced HU thresholds for both the lumbar spine (~200 HU) and femoral neck (~237 HU) in adult liver transplant recipients. These values are higher than those reported in non-contrast CT studies, reflecting the influence of contrast timing and protocol, and represent transplant- and protocol-specific reference ranges that may directly inform clinical surveillance strategies.

The high prevalence (45%) of reduced bone mass observed in our cohort is consistent with previous reports showing that patients with chronic liver disease and post-transplant immunosuppression are at substantial risk of osteoporosis [8,11,14]. Multifactorial mechanisms—including pre-transplant metabolic abnormalities, vitamin D deficiency, hypogonadism, and post-transplant exposure to glucocorticoids—contribute to accelerated bone loss [9]. Given the morbidity associated with fragility fractures, particularly in the early post-transplant period, practical and accessible tools for early skeletal evaluation are clinically valuable.

Previous studies evaluating bone health using CT have reported significant correlations between HU and DXA, ranging from r = 0.393 to 0.766 [19,20,25,26]. These findings support the potential utility of CT in assessing bone quality. However, the heterogeneity in correlation strength across studies may be explained by several methodological factors, including differences in CT acquisition protocols, reconstruction kernels, and ROI placement, all of which can introduce variability into HU measurements. Moreover, intrinsic limitations of DXA—such as its two-dimensional areal measurement, inability to distinguish trabecular from cortical components, and susceptibility to degenerative changes—may also contribute to inconsistencies [16]. For instance, Choi et al. [27] demonstrated that degenerative spinal changes substantially influence this relationship, reporting correlation coefficients of 0.398 in patients with degeneration and 0.734 in those without. In our study, we similarly observed significant correlations consistent with the existing literature. These results suggest the utility of CT-based opportunistic assessment of bone health in a population with LT.

In the literature, CT-derived HU measurements have demonstrated moderate to high diagnostic performance in identifying osteopenia and osteoporosis [28,29,30,31]. In our study, the diagnostic accuracy of HU values was consistent with previous studies, with AUCs ranging from approximately 0.700 for vertebral HU and up to 0.751 for femoral HU. The cut-off values—approximately 200 HU for vertebrae and 237 HU for the femur—were higher than those typically reported in non-contrast CT studies, reflecting our imaging conditions. Sensitivity and specificity values indicated a modest ability to distinguish between normal and low bone mass, whereas the relatively high NPV suggests that higher HU values are reassuring and can reliably exclude low bone mass in patients with elevated attenuation measurements. Although HU thresholds cannot replace formal densitometry, incorporating these simple measurements into routine abdominal CT reporting may help identify liver transplant recipients who would benefit from further evaluation, offering a practical and cost-free approach to strengthen osteoporosis screening in this high-risk population.

An additional observation in our study was the consistently higher HU values in the femur compared with the lumbar vertebrae. This expected pattern reflects the inherently greater cortical bone content of the proximal femur, which results in higher attenuation than the trabecular-rich lumbar spine [32]. Because trabecular bone is more metabolically active and undergoes osteoporotic changes earlier and more rapidly, lumbar vertebrae typically demonstrate lower HU values and are more sensitive to early bone loss [33]. This anatomical difference also aligns with our clinical findings: the prevalence of osteoporosis was lower at the femoral site and femoral DXA T-scores were generally higher than those of the lumbar spine.

Given that adult liver transplant recipients undergo frequent contrast-enhanced abdominal CT for routine graft and vascular surveillance, integrating HU measurements into this workflow may offer a practical triage strategy. Patients with clearly low HU values may be prioritized for timely DXA evaluation, metabolic work-up, and consideration of anti-osteoporotic therapy, whereas those with reassuring HU values may continue standard follow-up without immediate densitometric testing. Importantly, these HU measurements should be viewed as a complementary tool rather than a replacement for DXA or fracture-based risk assessment.

## 5. Limitations

This study is subject to several limitations. Primarily, the CT scans were acquired during the contrast-enhanced venous phase, which may influence HU measurements due to factors such as contrast timing, image reconstruction algorithms, and scanner-specific technical characteristics. Although these parameters may compromise inter-scan comparability, the standardized acquisition protocol and use of uniform scanner technology across the cohort likely reduced such variability. As the study was designed as a radiologically focused retrospective analysis, detailed clinical variables that influence bone metabolism were not uniformly available, which limits broader clinical interpretation and precludes fully adjusted multivariable modeling. Moreover, the cross-sectional design limits causal inference. Due to the retrospective design, phantom-based or phantom-free calibration required for formal quantitative CT densitometry could not be implemented; however, all CT systems used in this study undergo routine and mandatory calibration and quality assurance procedures in accordance with international standards and manufacturer guidelines to ensure HU stability in daily clinical practice. Finally, the two radiologists assessed different randomly assigned scan subsets; therefore, intra- and interobserver reproducibility could not be evaluated, representing a methodological limitation. Given these limitations, the findings of this study should be interpreted as preliminary and hypothesis-generating rather than definitive. Prospective, multicenter investigations with longitudinal follow-up are warranted to confirm and extend the present findings.

## 6. Conclusions

Abdominal CT examinations performed as part of routine post-transplant surveillance can be utilized for opportunistic assessment of skeletal attenuation using Hounsfield unit measurements. CT-derived HU values offer a practical and readily available approach for evaluating bone attenuation without additional imaging or radiation exposure. However, HU-based assessment should be regarded strictly as a complementary imaging metric and not as a substitute for established diagnostic modalities such as DXA. The present findings demonstrate the technical feasibility of HU-based bone attenuation analysis in liver transplant recipients; nevertheless, the results are preliminary, and further prospective studies incorporating clinical endpoints are required to clarify its role beyond methodological applicability.

## Figures and Tables

**Table 1:** Demographic and clinical characteristics of entire study cohort.

Variables	Categories	*n* (%)	Mean ± SD
Age (years)		259	55.68 ± 14.40
Age groups	<60	134 (51.7)	
	≥60	125 (48.3)	
Sex	Male	163 (62.9)	
	Female	96 (37.1)	
Lumbar spine BMD (g/cm^2^)		259	0.80 ± 0.13
Lumbar spine T-score		259	−2.37 ± 1.45
Lumbar T-score category	Normal	46 (17.8)	
	Osteopenia	95 (36.7)	
	Osteoporosis	118 (45.5)	
Femoral BMD (g/cm^2^)		259	0.85 ± 0.18
Femoral T-score		259	−0.74 ± 1.24
Femoral T-score category	Normal	186 (71.8)	
	Osteopenia	46 (17.8)	
	Osteoporosis	27 (10.4)	55.68 ± 14.40

**Table 2:** Comparison of quantitative CT and DXA parameters between patients aged <60 years and ≥60 years.

Variables	<60 Years (*n* = 134)	≥60 Years (*n* = 125)	Mean Difference (95% CI)	*p*
Sagittal mean HU	207 ± 52.5	164 ± 44.8	43.6 (31.6–55.6)	<0.001
Axial mean HU	208 ± 80.5	162 ± 44.5	45.9 (29.8–62.0)	<0.001
Femoral HU	313 ± 69.2	277 ± 68.8	36.7 (19.8–53.6)	<0.001
Lumbar BMD (g/cm^2^)	0.84 ± 0.13	0.76 ± 0.14	0.08 (−0.05–0.21)	0.208
Lumbar T-score	−2.29 ± 1.62	−2.46 ± 1.24	0.17 (−0.19–0.52)	0.355
Femoral BMD (g/cm^2^)	0.85 ± 0.18	0.85 ± 0.17	0.00 (−0.04–0.05)	0.884
Femoral T-score	−0.70 ± 1.32	−0.78 ± 1.16	0.08 (−0.23–0.38)	0.618

**Table 3:** Comparison of mean vertebral HU values among lumbar T-score groups.

Parameters	Groups	Mean ± SD	95%CI for Mean	*p*	Post Hoc	*p*	Mean Difference (95%CI)
Sagittal HU	Normal (*n* = 46)	216 ± 54.3	200–233	<0.001	0 vs. 1	0.080	19.3 (−1.8–40.3)
	Osteopenia (*n* = 95)	197± 45.1	188–206		0 vs. 2	<0.001	51.3 (30.9–71.6)
	Osteoporosis (*n* = 118)	165 ± 51.3	156–175		1 vs. 2	<0.001	32.0 (15.8–48.13)
Axial HU	Normal (*n* = 46)	214 ± 55.5	197–230	<0.001	0 vs. 1	0.453	14.3 (−13.8–42.5)
	Osteopenia (*n* = 95)	200 ± 86.3	182–217		0 vs. 2	<0.001	51.9 (23.7–78.1)
	Osteoporosis (*n* = 118)	163 ± 49.7	154–172		1 vs. 2	<0.001	36.6 (15.0–58.2)
Femoral HU	Normal (*n* = 186)	307 ± 69.7	297–317	<0.001	0 vs. 1	0.040	27.6 (0.96–54.2)
	Osteopenia (*n* = 46)	279 ± 68.9	259–300		0 vs. 2	<0.001	68.3 (33.2–103.3)
	Osteoporosis (*n* = 24)	239 ± 58.5	214–263		1 vs. 2	0.051	40.7 (−0.09–81.4)

0: Normal; 1: Osteopenia; 2: Osteoporosis.

**Table 4:** Receiver operating characteristic analysis of CT-derived HU measurements for identifying reduced bone mineral density.

Groups	Parameters	AUC (95% CI)	*p*	Cutt-Off (HU)	Sens	Spec	PPV	NPV
Normal vs. (Osteopeni + Osteoporosis)	Sagittal mean HU (L1–L4)	0.68 (0.60–0.76)	<0.001	203	0.63	0.68	0.29	0.89
	Axial mean HU (L1–L4)	0.69 (0.60–0.77)	<0.001	205	0.63	0.70	0.28	0.89
	Femoral HU	0.67 (0.60–0.75)	<0.001	240	0.83	0.57	0.79	0.63
(Normal + Osteopenia) vs. Osteoporosis	Sagittal mean HU (L1–L4)	0.70 (0.64–0.76)	<0.001	198	0.64	0.67	0.69	0.63
	Axial mean HU (L1–L4)	0.70 (0.64–0.76)	<0.001	200	0.62	0.66	0.67	0.61
	Femoral HU	0.75 (0.65–0.85)	<0.001	237	0.82	0.63	0.81	0.65

## Data Availability

The datasets analyzed during the current study are available from the corresponding author on reasonable request.
